# Evaluation and validation of extensive growth and growth boundary models for mesophilic and psychrotolerant *Bacillus cereus* in dairy products (Part 2)

**DOI:** 10.3389/fmicb.2025.1553903

**Published:** 2025-03-31

**Authors:** Maryam Maktabdar, Rannvá Høgnadóttir Houmann, Nanna Hulbæk Scheel, Karoline Broskov Skytthe, Ellen Wemmenhove, Elissavet Gkogka, Paw Dalgaard

**Affiliations:** ^1^Food Microbiology and Hygiene, DTU National Food Institute (DTU Food), Technical University of Denmark, Kgs. Lyngby, Denmark; ^2^Arla Foods Ingredients Innovation Center, Arla Foods Ingredients, Videbæk, Denmark; ^3^Arla Innovation Center, Arla Foods amba, Aarhus, Denmark

**Keywords:** predictive modelling, model validation, product formulation, microbial risk assessment, food safety

## Abstract

Performance was evaluated for two extensive models to predict growth and growth boundaries of mesophilic and psychrotolerant *Bacillus cereus* in dairy products. Both models incorporated the inhibitory effect of 11 environmental factors and of their interactions. The two models were calibrated and evaluated using data from 66 and 67 new challenge tests, respectively, conducted with various types of well-characterized dairy products. Additionally, the mesophilic model was evaluated using 139 growth responses from literature (growth/no-growth, lag time, and *μ_max_* values) for 24 different *B. cereus* strains. The psychrotolerant model was evaluated using 109 growth responses from published studies and including data for 26 strains in dairy products. The predictive performance of the evaluated models was compared with four existing models for mesophilic *B. cereus* and four different models for psychrotolerant *B. cereus*. The new mesophilic model had good performance and predicted growth responses in new challenge tests, with bias-/accuracy-factor values of 1.13/1.49 and 80% correct, 17% fail-safe, and 3% fail-dangerous growth/no-growth predictions. With literature data for mesophilic *B. cereus*, predictions were good with bias-/accuracy-factor values of 0.97/1.36 and 91% correct, 9% fail-safe, and 0% fail-dangerous predictions. The evaluated psychrotolerant model also exhibited good performance in predicting growth responses for new challenge tests, with bias-/accuracy-factor values of 1.07/1.38 and 84% correct, 14% fail-safe, and 2% fail-dangerous predictions for growth/no-growth responses. With literature data for psychrotolerant *B. cereus*, this model did not acceptably predict growth rates at temperatures <10°C. Therefore, the temperature term of the model was expanded at temperatures from 1°C to 10°C. The performance of the updated psychrotolerant model was markedly improved, achieving bias-/accuracy-factor of 1.07/1.80, and 91% correct, 9% fail-safe, and 0% fail-dangerous predictions. The two new and extensive models offer significant advantages over existing models by including the growth inhibiting effects of more environmental factors and their interactions, resulting in un-biased predictions for a wider range of dairy matrices. These validated models can support management of mesophilic and psychrotolerant *B. cereus* growth in diverse dairy products, contribute to risk assessments and to optimization of combinations of relevant growth-inhibitory factors during product formulation and innovation.

## 1 Introduction

*Bacillus cereus sensu lato* is a diverse group of spore-forming pathogens classified as psychrotolerant, mesophilic, or thermophilic groups based on their temperature growth responses (Guinebretière et al., 2008; Carlin et al., 2013). The prevalence of *B. cereus s.l.* in dairy products can be high with concentrations of up to about 100 CFU/g or CFU/mL, making it important to limit or prevent their growth (EFSA, 2005; EC, 2007; Tirloni et al., 2022; Maktabdar et al., 2024). However, guidelines for combined product characteristics and storage conditions to prevent unacceptable growth of *B. cereus s.l.* in foods, including dairy products, are lacking. Validated growth and growth boundary models are therefore valuable tools to predict and manage *B. cereus* responses in dairy products and other foods. Several models are available to predict growth responses of psychrotolerant, mesophilic, or thermophilic *B. cereus*, using either cardinal parameter or polynomial equations. Secondary growth models incorporating the effect of temperature alone or in combination with one to three factors, such as pH, water activity (a_w_), CO_2_, lactic acid or nitrite were developed using laboratory broth or dairy matrices (Benedict et al., 1993; Sutherland et al., 1996; Zwietering et al., 1996; Valík et al., 2003; Ölmez and Aran, 2005; Carlin et al., 2013; Buss da Silva et al., 2017; Juneja et al., 2019; Ellouze et al., 2021; Le Marc et al., 2021a,b; Park et al., 2021; Sarkar et al., 2023). Recently, more extensive secondary growth models were developed using laboratory broth. For psychrotolerant *B. cereus*, Le Marc et al. (2024) suggested growth boundary models incorporating the effect of temperature, pH, a_w_, acetic, and lactic acids. For mesophilic and psychrotolerant *B. cereus* from dairy products, Maktabdar et al. (2025; Part 1) developed two growth and growth boundary models. These new models were more extensive than previously developed models and incorporated the combined effect of 11 environmental factors.

Predictive food microbiology models must be reasonably accurate in predicting the growth responses of pathogens in food to be useful for evaluating and managing product safety. These models should include the effect of environmental factors that significantly influence growth in targeted products and they should be validated for those specific foods (Couvert et al., 2010; Mejlholm et al., 2010). For dairy products, the performance of available *B. cereus* growth models has been primarily evaluated for milk and reconstituted infant formula (Zwietering et al., 1996; Valík et al., 2003; Buss da Silva et al., 2017), with limited data available for other dairy products. This represents an important challenge for applying these available models to accurately predict *B. cereus* growth in other dairy matrices. For *B. cereus* and other bacteria, including lactic acid bacteria, *Listeria monocytogenes*, psychrotolerant pseudomonas, and *Clostridium sporogenes*, product validation studies have shown that growth rates in laboratory broth can differ from those in specific dairy products, even when environmental conditions for the two matrices appear similar (Østergaard et al., 2014; Martinez-Rios et al., 2016; Buss da Silva et al., 2017; Koukou et al., 2022b). Therefore, growth models developed using laboratory broth may need calibration to provide un-biased growth rate predictions in targeted food products. For cardinal parameter models, the *μ_opt_* or *μ_ref_* parameter values can be calibrated to make broth models realistic for various types of food (Østergaard et al., 2014; Martinez-Rios et al., 2016; Koukou et al., 2022a). Buss da Silva et al. (2017) calibrated a *B. cereus* growth model, developed using broth, with a factor of 0.67 to give un-biased prediction of growth rates for reconstituted infant formula.

The present study evaluated and validated the performance of two extensive growth and growth boundary models of mesophilic and psychrotolerant *B. cereus*. These models were developed by Maktabdar et al. (2025; Part 1) using liquid laboratory broth and a ultra filtration (UF) permeate of whey. A comprehensive dataset with growth responses and dairy product characteristics was generated using new challenge tests performed as part of the present study and data from previously published studies with dairy products. This dataset was used to evaluate and determine (i) lag times including potential differences between vegetative cells and spores, (ii) the need for calibration of *μ_opt_* to obtain un-biased predictions of growth rates in dairy matrices of interest, and (iii) the models range of applicability where they have been successfully validated with respect to types of dairy matrices, product characteristics, and storage temperatures. Additionally, the performance of the new and extensive models was compared with other existing models to predict growth responses of mesophilic or psychrotolerant *B. cereus* in dairy products.

## 2 Materials and methods

### 2.1 Strain cocktails and pre-cultures

Growth responses of two cocktails of vegetative mesophilic (Mix-Bc_mes_) or psychrotolerant (Mix-BC_psy_) *B. cereus* isolates were studied in challenge tests with dairy products. Mix-Bc_mes_ and Mix-BC_psy_ included, respectively, six and seven potentially pathogenic isolates. These isolates were previously obtained from dairy products and used for the development of two new and extensive models to predict growth and growth boundary of mesophilic or psychrotolerant *B. cereus* (Maktabdar et al., 2024). Prior to inoculation of challenge tests, isolates were cultured and strain cocktails prepared as previously described (Maktabdar et al., 2024, 2025; Part 1).

### 2.2 New challenge tests for product evaluation of models

Commercially available dairy products (brie, danbo, mascarpone, mozzarella, processed cheese, ricotta, rice pudding, semi-skimmed milk, tiramisu) and a dairy alternative (oat drink) were purchased during 2023–2024 from local supermarkets (*n* = 29). Additionally, customized dairy products were produced by Arla Foods (mascarpone) or at DTU Food (processed cheese and paneer). Dairy ingredient solutions including liquid whey, concentrated whey products, and solutions made from various powders provided by Arla Foods Ingredients were also used in inoculated challenge tests and in un-inoculated control treatments as described below. Un-inoculated control treatments were performed for all challenge tests to evaluate if the observed growth responses of *B. cereus* resulted from the inoculated strain cocktails rather than naturally occurring *B. cereus* in the studied dairy matrices. The un-inoculated control treatments were not designed to study growth kinetics of naturally occurring *B. cereus* and these experiments were stopped when *B. cereus* in inoculated treatments reached their stationary phase. However, for some un-inoculated control treatments kinetics of the naturally occurring *B. cereus* were quantified.

#### 2.2.1 Customized cream cheese (mascarpone)

Mascarpone produced by Arla Foods was used to formulate different recipes of cheeses (mascarpone 1–9; Supplementary Table S1) with desired concentrations of organic acids and NaCl. Different recipes of mascarpone cheese were used in 21 challenge tests with Mix-Bc_mes_ or Mix-Bc_psy_. The desired pH was adjusted with HCl or NaOH (Table 1 and Supplementary Table S1). For all commercial and customized cheeses, pH was measured with a direct pH measurement probe for solid food (Section 2.5).

**Table 1:** Range of product characteristics and growth kinetics of Mix-BC_mes_ or Mix-Bc_psy_ in new challenge tests used for model evaluation^a^.

n^b^	Temp (°C)	pH	WPS^c^ (%)	Organic acids in water phase (ppm)					Phosphate ions^d^ (%)			*RLT* ^e^	*μ_max_*^f^ (h^−1^)
				Acetic	Citric	Lactic	Benzoic	Sorbic	P1	P2	P3		
Commercial products incl. brie, danbo cheese, mascarpone, milk, mozzarella, oat drink, processed cheese, rice pudding, ricotta, tiramisu													
27	3–24	5.5–7.3	0.1–2.7	0–1,534	831–3,539	0–13,410	ND^g^	ND	0.0–2.3	ND	ND	0–29	0.00–0.31
Customized processed cheese													
28	15–18	5.2–5.8	0.8–3.0	25–1,490	1,542–8,126	0–17,472	ND	ND	0.0–3.1	0–1.7	0–2.1	0–29	0.00–0.17
Customized cream cheese, mascarpone, and paneer													
27	13–24	4.9–5.7	0.4–4.6	0–509	1,086–2,595	0–6,221	0–537	0–566	ND	ND	ND	0–15	0.00–0.49
Solutions from dairy ingredients													
41	8–39	5.3–7.3	0.0–5.1	0–459	0–4,215	0–3,624	ND	ND	ND	ND	ND	0–20	0.00–1.62

a Detailed information is provided for individual studies in Supplementary Table S1.

b Number of challenge tests performed with Mix-Bc_mes_ or Mix-Bc_psy_.

c Water phase salt (%, w/w).

d Concentration of phosphate ions in water phase (%, w/w).

e Relative lag time (RLT) calculated for experiments where growth was observed.

f Fitted maximum specific growth rates (μ_max_).

g Not detected (ND).

#### 2.2.2 Customized processed cheese

Different processed cheese recipes were produced using either dairy powders or mascarpone (Arla Foods) as the base formulation (PC 1–15; Table 1 and Supplementary Table S1). Processed cheese based on dairy powders was produced by mixing milk protein (Nutrilac, Arla Foods), skimmed milk powder (Arla Foods), cheese powder (Lactosan, Denmark), unsalted butter, and water as previously reported by Koukou et al. (2022b). Recipes were customized with different concentrations of organic acids, NaCl, and phosphate salts (*n* = 26; Table 1 and Supplementary Table S1). The cheese mixture was heated at 80°C for 10 min in a thermomixer (Thermomix TM5, Vorwerk, Wuppertal, Germany). Then pH of the chilled cheese was adjusted to the desired level with HCl or NaOH (Table 1 and Supplementary Table S1). Processed cheese based on mascarpone (Arla Foods) was produced by adding organic acids, NaCl, and phosphate melting salts at desired concentrations (Table 1 and Supplementary Table S1). This cheese was also heat treated at 80°C for 10 min, and its pH was adjusted using HCl or NaOH (PC 12, and PC 14; *n* = 4).

#### 2.2.3 Customized paneer

Different recipes of paneer were produced by using skimmed milk, semi-skimmed milk, and whole milk (*n* = 6; Paneer 1–5; Supplementary Table S1). The milk was heated and kept at 90°C for 120 s. HCl was used to obtain pH values of 5.2–5.4. The milk coagulated for 10 min. Then, the excess whey was removed by using a cheese cloth. Desired concentrations of organic acid and NaCl were then added to the cheese (Table 1 and Supplementary Table S1).

#### 2.2.4 Dairy ingredients solutions

Liquid thin whey and concentrated whey products including whey protein concentrates (WPC35 with 35% protein in dry matter, WPC and Bacteria-filtered WPC) as well as reverse osmosis concentrate (RO) without customization were used for challenge tests (*n* = 10; Table 1 and Supplementary Table S1; Arla Food Ingredients). Customized dairy solutions were prepared by mixing different concentrations of dairy powders (WPC, Bacteria-filtered WPC, UF permeate or whey fat concentrate [WFC]; Arla Foods Ingredients) with demineralized water. Desired concentrations of NaCl were added and pH was adjusted with HCl or NaOH to desired levels (*n* = 36). High and low concentrations of UF permeate, and WFC solutions (different dry matter content) were tested to analyze the effect of nutrient availability on growth rates (UF permeate 1 and 3; WFC solutions 1, 2 and 3; Supplementary Table S1).

### 2.3 Challenge tests and primary modelling

Dairy products and solutions of dairy ingredients were inoculated with 0.1% (v/w) of Mix-Bc_mes_ or Mix-Bc_psy_ pre-cultures to obtain initial concentrations of around 3 log CFU/g or CFU/mL. The inoculum was thoroughly mixed into liquid or semi-solid dairy products. For solid matrices like danbo or mozzarella cheeses, the cheese was shredded into approximately 0.5 cm pieces prior to inoculation to ensure a homogenous distribution of the inoculum. Solid samples were distributed in multiple 100 mL closed-lid containers (Combi Tp95-180, Deca, Belgium), while liquid samples were stored in multiple glass bottles. All samples were stored under aerobic conditions at different temperatures (Table 1 and Supplementary Table S1). Storage temperature for each challenge test was recorded every 30 min in duplicate by data loggers (TinytagPlus, Gemini Data Loggers Ltd., Chichester, UK). Challenge tests were terminated when *B. cereus* was well into the stationary growth phase or in a few cases due to visible growth of molds on products. For viable counting of solid foods, duplicate 10 ± 1 g samples from different containers were diluted 10-fold with physiological peptone water (0.85% NaCl and 0.1% Bacto peptone, 211677, BD Bioscience, San Jose, USA). The diluted samples were homogenized for 1 min at normal speed in a stomacher (Stomacher 400 Circulator, Seward Medical, London, UK). For liquid foods, 1 mL in two replicates from different bottles was used to prepare required 10-fold dilutions in physiological peptone water. Growth of aerobic viable count (AVC) and *B. cereus* were enumerated during product storage by surface plating. Brian Heart Infusion (BHI) agar (Oxoid, CM1136B, Hampshire, UK) was used for AVC (25°C; 48 h), and mannitol egg yolk polymyxin (MYP) agar (SSI Diagnostica, Hillerød, Denmark) was used for selective enumeration of *B. cereus* (30°C; 24 h). Concentrations of bacteria were expressed as log CFU/g or log CFU/mL and duplicate counts at each sampling time in growth experiments were fitted with the integrated and log transformed logistic model with or without delay (Equation 1). Thus, for each challenge test one growth rate was estimated from duplicate counts during the storage time.


(1) $$\begin{array}{l} \log\left(N_{t}\right)\\ =\{\begin{array}{c} \log\left(N_{0}\right),\;t<t_{\mathrm{lag}}\\ \log\left(\frac{N_{\text{max}}}{1+\left(\frac{N_{\text{max}}}{N_{0}}-1\right)\cdot \exp\left(-\mu_{\text{max}}\cdot \;\left(t-t_{\mathrm{lag}}\right)\right)}\right),\;t\geq t_{\mathrm{lag}} \end{array} \end{array}$$

where *N_0_* and *N_max_* are the fitted initial and maximum cell concentrations (CFU/g or CFU/mL), respectively. *μ_max_* is the maximum specific growth rate (h^−1^), *t* is storage time (h), and *t_lag_* is the lag time (h). An F-test was used to determine if *t_lag_* was significant with *p* value below 0.05.

### 2.4 Relative lag time (*RLT*) and maximum population density (*N_max_*)

The *t_lag_* and *μ_max_* values from each individual challenge test (Supplementary Table S1) were used to determine the relative lag time (*RLT*; Equation 2; Mellefont and Ross, 2003). It was determined whether *RLT* was dependent on storage temperature (*RLT* = *K1* + *K2*/*T*^2^) or constant (*RLT* = *K1*) as reported by Hereu et al. (2014).


(2) $$RLT=\frac{t_{\mathrm{lag}}.\;\mu_{\text{max}}}{\ln\left(2\right)}$$

It was assessed whether log(*N_max_*) values, as determined for new challenge tests, were constant or dependent on storage temperature (Equation 3). In this equation b1 and b2 are constant, and T is the storage temperature.


(3) $$\log\left(N_{\text{max}}\right)=b_{1}-\frac{b_{2}}{T^{2}}$$

### 2.5 Product characteristics

For each challenge test, product characteristics were determined in duplicate prior to inoculation and storage. The dry matter content was determined by oven drying at 105°C for 22 ± 2 h. NaCl concentration was measured by automated potentiometric titration (785 DMP Titrino, Metrohm, Hesisau, Switzerland), as described previously (Martinez-Rios et al., 2016; Koukou et al., 2021). For liquid foods and solutions, pH was measured with a PHM 250 Ion Analyzer (MetroLab™, Radiometer, Copenhagen, Denmark). For solid/semi-solid foods, pH was measured with a pH probe (HACH, PHC108, US), specific for direct pH measurement. Concentrations of benzoic, citric, lactic, and sorbic acids in samples were determined using HPLC with external standards for both identification and quantification (Martinez-Rios et al., 2019). An enzymatic acetic acid assay (Acetic acid kit K-ACET, Megazyme, Ireland) was used to quantify the concentration of acetic acids in duplicate for each challenge test. Lipid or fat content was measured in duplicate for nine selected products (paneer 1–5; mascarpone 4, 5, 8, and 9; Supplementary Table S1). The measurements were performed by Eurofins, Denmark, using a gravimetry method (ISO 1735:2004/IDF 5), or at DTU Food using the method of Bligh and Dyer (1959). These fat content data were used to calculate the concentration of benzoic and sorbic acid in the water phase of products as previously described (Equation 4; Brocklehurst and Wilson, 2000; Cheng et al., 2010; Koukou et al., 2022b; Mejlholm and Dalgaard, 2015).


(4) $$OA_{wp}=\frac{OA_{p}.\;100}{\left[1+K_{p}\left(\frac{\theta }{1-\theta }\right)]\cdot \;(100-DM+\mathrm{Fat}\right)}$$

where *OA_wp_* is the concentration of benzoic or sorbic acids in water phase (ppm), *OA_p_* is the total concentration of organic acids in the product (ppm), *DM* is the dry matter concentration (%), and *Fat* is fat content (%). *θ* is the fraction of fat in the water and fat phase, and K_p_ is the partition coefficient between water and fat which was set to 7.22 and 4.19 for benzoic and sorbic acid, respectively.

Concentrations of mono-, di-, and tri-phosphate were estimated from recipes for relevant samples (*n* = 26).

### 2.6 Collection of growth responses from literature data for evaluation of models

Reported *B. cereus* growth responses in dairy products were collected from published studies. When growth rates were not explicitly provided, the WebPlotDigitizer v. 4 software^1^ was used to extract cell concentration data from growth curves. Then, growth rates were estimated by fitting Equation 1 to these extracted growth curve data. If mesophilic or psychrotolerant characteristics of isolates were not reported, growth at temperatures below 7°C was considered as psychrotolerant growth. One data point regarding growth of a mesophilic strains cocktail at 50°C (Sarkar et al., 2023) was excluded from the model evaluation, as it exceeded the models *T_max_* values. For studies where organic acid concentrations were not reported then levels were assumed from known concentrations in similar products (Aragon-Alegro et al., 2007; Tirloni et al., 2019; Wemmenhove et al., 2021). If this was not possible then the effect of organic acids was not considered, i.e., the organic acid concentration was assumed to be zero, to ensure fail-safe predictions.

### 2.7 Calibration and evaluation of the two new and extensive models

Due to good performance of the mesophilic model no product-calibration of its *μ_opt_* value was performed. Product-calibration was exclusively performed for the psychrotolerant model to better reflect the growth rates observed in dairy products. The *μ_opt_* value of the psychrotolerant model was calibrated using challenge test data from dairy products. For this purpose, 10 observed *μ_max_* values from Mix-Bc_psy_ in different dairy products including brie, mascarpone, processed cheese, RO, thin whey, and a UF permeate (Table 2 and Supplementary Table S1) were compared with the *μ_max_* values predicted by Equation 5 using cardinal parameters values and a *μ_opt_* value of 2.12 h^−1^ as determined by Maktabdar et al. (2025; Part 1) and shown in Supplementary material for the present manuscript.


(5) $$\begin{array}{c} \mu_{\text{max}}=\mu_{\mathrm{opt}}\cdot CM\left(T\right)\cdot CM\left(pH\right)\cdot CM\left(a_{w}\right)\cdot CM\left(\mathrm{AA}C_{u}\right)\cdot \\ CM\left(\mathrm{BA}C_{u}\right)\cdot CM\left(CAC_{T}\right)\cdot CM\left(LAC_{u}\right)\cdot \\ CM\left(SAC_{u}\right)\cdot CM\left(P_{1}\right)\cdot CM\left(P_{2}\right).\cdot CM\left(P_{3}\right)\cdot \xi \end{array}$$

where *μ_opt_* is the maximum specific growth rate at optimum temperature and pH 6.00 ± 0.05. *CM* represents the cardinal model terms for the effects of temperature (T), pH, a_w_, undissociated acetic, benzoic, lactic, and sorbic acids (AAC_u_, BAC_u_, LAC_u_, and SAC_u_, respectively), total citric acid (CAC_T_), and ortho-, di-, and tri-phosphate (P_1_, P_2_, and P_3_, respectively). *ξ* express the quantitative effect, from zero to one, of interaction between the *CM* terms. The value of the interaction term *ξ* is determined from the parameter *ψ* which expresses how far a set of specific environmental factors are from the predicted growth boundary with *ψ* = 1.0.

**Table 2:** Range of product characteristics and growth kinetics of Mix-Bc_psy_ in new challenge tests used for calibration of psychrotolerant model^a^.

n^b^	Temp (°C)	pH	WPS^c^ (%)	Organic acids in water phase (ppm)			Phosphate ions^d^ (%)			*RLT* ^e^	*μ_max_*^f^ (h^−1^)
				Acetic	Citric	Lactic	P1	P2	P3		
Commercial products incl. brie and mascarpone cheeses											
2	15	6.3–6.8	0.4–2.4	0–208	0–2,437	0–1,468	ND^g^	ND	ND	0	0.16-0.33
Customized processed cheese											
2	15	5.2–5.6	1.0–2.2	82–129	1,916–2,097	580–612	0–3.0	0–0.5	ND	1–4	0.11–0.18
Customized mascarpone											
1	15	5.5	0.4	82	2,183	525	ND	ND	ND	2	0.22
Solutions from dairy ingredients											
5	8–15	5.7–7.3	0.1–0.6	0–65	0–4,215	0–3,624	ND	ND	ND	6–20	0.05–0.78

a Detailed information is provided for individual studies in Supplementary Table S1.

b Number of challenge tests performed with Mix-Bc_psy_ used for psychrotolerant model calibration.

c Water phase salt (%, w/w).

d Concentration of phosphate ions in water phase (%, w/w).

e Relative lag time (RLT) calculated for experiments where growth was observed.

f Fitted maximum specific growth rates (μ_max_).

g Not detected (ND).

Bias (B_f_) and accuracy (A_f_) factor values for these observed and predicted *μ_max_* values were calculated, as explained below, and calibration was performed by using Equation 6 (Koukou et al., 2021).


(6) $$\mu_{\mathrm{opt}-\mathrm{cal}}=\frac{\mu_{\mathrm{opt}}}{B_{f}}$$

where *μ_opt-cal_* is the calibrated maximum specific growth rate for dairy products at the optimum temperature for growth.

The performance of the mesophilic and of the calibrated psychrotolerant models was evaluated by comparing predicted and observed growth responses. B_f_ and A_f_ factor values based on the *μ_max_* data and percentage of correct, fail-safe (growth predicted but not observed) and fail-dangerous (growth observed but not predicted) were calculated (Ross, 1996; Mejlholm et al., 2010). B_f_ values between 0.95 and 1.11 indicate good model performance, values between 0.87 and 0.95 or 1.11 and 1.43 indicate acceptable performance, and values outside this range suggest unacceptable model performance (Mejlholm et al., 2010). A_f_ values above 1 + 0.15x number of environmental factors in model were considered unacceptable (Ross et al., 2000). Growth boundary predictions were considered acceptable if the models achieved a correct growth/no-growth prediction of 75–80% or higher, had fail-dangerous predictions below 5%, and fail-safe predictions below 15–20%. Growth was defined as an increase of >0.5 log CFU/g or log CFU/mL (Mejlholm et al., 2010).

Evaluation of the mesophilic and calibrated psychrotolerant models was conducted using data from 66 and 57 challenge tests with Mix-Bc_mes_ and Mix-Bc_psy_, respectively, from the present study (Table 1 and Supplementary Table S1). Additionally, 100 growth responses of mesophilic *B. cereus* from literature studies, were used for evaluation of the mesophilic model (Table 3), 70 responses for psychrotolerant *B. cereus* were used for evaluation of the psychrotolerant model (Table 4), and 39 responses for isolates, without a clear classification as mesophilic or psychrotolerant, were used for evaluation of both models (Table 5).

**Table 3:** Product characteristics and growth kinetics of mesophilic *B. cereus* strains from literature data used for mesophilic model evaluation^a^.

Products	n	Temp (°C)	pH	WPS^b^ %	Organic acid in water phase (ppm)			*RLT* ^c^	*μ_max_* (h^−1^)	Reference
					Acetic	Citric	Lactic			
Fresh cheese	4	15	5.1–6.9	2.18	982–1,092	2,226–2,575	42–1,474	NR^d^	0.25-0.50	Tirloni et al. (2020)
Mascarpone, Milk, Taleggio cheese, Yogurt	10	15–37	4–6.7	0.5–6.5	**0-189** ^**e**^	**0-2,400**	**0-11,606**	6–12	0.00–0.22	Tirloni et al. (2017b)
Milk	1	30	**6.7**	**0.5**	**0**	**0**	**0**	4	0.61	Bartoszewicz et al. (2013)
Paneer	8	10–45	5.5	0.13	**0**	**0**	**0**	1–5	0.05–1.70	Sarkar et al. (2023)
Past./fermented milk, fruit milk	5	30	3.6–6.5	**0.5**	**0**	**0**	**0**	7–10	0.00–1.94	Wong et al. (1988)
Primo Sale fresh cheese	1	15	6.4	**2.2**	**0**	**0**	**0**	–	0.00	Tirloni et al. (2021)
Reconstituted infant formula	10	8–24	5.8–6.7	**0.5**	**0**	**0**	**0**	3–12	0.00–1.25	Bursová et al. (2018)
Reconstituted infant formula	45	9–25	**6.7**	**0.5**	**0**	**0**	**0**	0–12	0.00–0.95	Buss da Silva et al. (2017)
Reconstituted milk	7	12–45	6.8	0.8	**0**	**0**	**0**	6	0.11–1.54	Ellouze et al. (2021)
Reconstituted milk, infant formula, milk	3	20–30	**6.7**	**0.5**	**0**	**0**	**0**	NR	0.32–1.5	Sutherland et al. (1996)
Ricotta	6	10–15	6.0	**0.5**	**0**	**3,393**	**291**	4–9	0.04–0.29	Tirloni et al. (2017a)

a Detailed information is provided for individual studies in Supplementary Table S2.

b Water phase salt (w/w, %).

c Relative lag time (RLT).

d Not reported (NR).

e Values assumed from similar products are shown with bold font.

**Table 4:** Product characteristics and growth kinetics of psychrotolerant *B. cereus* strains from literature data used for psychrotolerant model evaluation^a^.

Product	n	Temp (°C)	pH	WPS^b^ %	Organic acid in water phase (ppm)			*RLT* ^c^	μ_max_ (h^−1^)	Reference
					Acetic	Citric	Lactic			
Brie	4	8–20	6.8	3.8	**0**	**0**	**1,468**	0	0.00–0.58	Little and Knøchel (1994)
Carbonated/non-carbonated fermented bifidus milk	2	37	6.0–6.7	**0.5** ^d^	270-290	**0**	143-195	0	0.94–1.57	Noriega et al. (2003)
Milk	2	7–30	**6.7**	**0.5**	**0**	**0**	**0**	0–11	0.07–0.28	Bartoszewicz et al. (2013)
Past. milk	6	5–13	**6.7**	**0.5**	**0**	**0**	**0**	2–16	0.03–0.25	Valík et al. (2003)
Reconstituted skim milk	4	10	5.0–6.7	**0.5–**3.0	**0**	**0**	**0**	0	0.00–0.05	Sadek et al. (2006)
Skim milk broth	6	4–6	**6.5**	**0.5**	**0**	**0**	**0**	13–71	0.05–0.14	Buehler et al. (2018)
Skim milk broth	2	6	**6.7**	**0.5**	**0**	**0**	**0**	1	0.00–0.06	Ivy et al. (2012)
Chilled dairy model, chocolate mousse, Vanilla dessert	12	8–12	6.0–6.5	**0.5–1.7**	**0**	**0**	**0**	0–28	0.00–0.13	O’Mahony et al. (2001)
Ricotta salata	1	4	6.2	4.45	**0**	**3,393**	**291**	–	0.00	Spanu et al. (2016)
Semi-skimmed milk, milk	22	6–10	**6.7**	**0.5**	**0**	**0**	**0**	1–83	0.01–0.26	Zwietering et al. (1996)
Ultrafiltered feta cheese	9	4	4.7–5.1	3.3–4.6	**0**	**0**	**0**	–	0.00	Moradi-Khatoonabadi et al. (2015)

a Detailed information is provided for individual studies in Supplementary Table S2.

b Water phase salt (w/w, %).

c Relative lag time (RLT).

d Values assumed from similar products are shown with bold font.

**Table 5:** Product characteristics and growth kinetics of *B. cereus* strains from literature data for strains with unknown mesophilic or psychrotolerant characteristics; used for evaluation of both mesophilic and psychrotolerant models.^a^

Products	n	Temp (°C)	pH	WPS^b^ %	Organic acid in water phase (ppm)			*RLT* ^c^	*μ_max_* (h^−1^)	Reference
					Acetic	Citric	Lactic			
Gouda cheese	1	12–15	5.2	4.7	**0** ^d^	**0**	**36,567**	–	0.00	Rukure and Bester (2001)
Milk	3	15–30	**6.7**	**0.5**	**0**	**0**	**0**	3–5	0.25–1.55	Kim et al. (2013)
Past. & raw cow/goat/sheep milk	8	8–22	**6.5–6.7**	**0.5**	**0**	**0**	**0**	0–27	0.00–0.86	Necidová et al. (2014)
Reconstituted infant food	5	27	**6.7**	**0.5**	**0**	**0**	**0**	5–14	1.08–2.15	Becker et al. (1994)
Reconstituted infant formula	20	8–24	5.8–6.7	**0.5**	**0**	**0**	**0**	3–12	0.00–1.25	Bursová et al. (2018)
UHT milk	2	20–37	**6.7**	**0.5**	**0**	**0**	**0**	NR^d^	0.39–1.55	De Jonghe et al. (2010)

a Detailed information is provided for individual studies in Supplementary Table S2.

b Water phase salt (w/w; %).

c Relative lag time (RLT).

d Values assumed from similar products are shown with bold font.

### 2.8 Existing models to predict growth responses for mesophilic or psychrotolerant *B. cereus*

Available predictive models evaluated in the present study include four models for vegetative mesophilic *B. cereus* cells (Ölmez and Aran, 2005; Carlin et al., 2013; Ellouze et al., 2021; Le Marc et al., 2021a) and psychrotolerant models from four studies (Zwietering et al., 1996; Carlin et al., 2013; Le Marc et al., 2021a, 2024). These models included the effect of from one to five environmental factors (Tables 6, 7). An overview of these models, including equations, and specific parameter values is provided in Supplementary material.

**Table 6:** Evaluation of the new and existing models for mesophilic *B. cereus* by using new challenge tests and literature data.

Model	Maktabdar et al. (2025; Part 1)		Carlin et al. (2013)		Le Marc et al. (2021a)		Ellouze et al. (2021)		Ölmez and Aran (2005)	
Environmental factors included in model	Temp, pH, a_w_, acetic, benzoic, citric, lactic, sorbic acids, ortho-, di-, and tri-phosphates		Group IV strain F4430/73 Temp, pH, a_w_		Group III strain B648 Temp, pH		Group III emetic strain F4810/72 Temp		Temp, pH, a_w_, LAC	
Datasets	CT^a^	LD^b^	CT	LD	CT	LD	CT	LD	CT	LD
n^c^	47	122	49	120	48	116	49	121	49	121
B_f_	1.13	0.97	1.27	1.16	1.42	1.10	1.03	0.62	0.95	1.30
A_f_	1.49	1.36	1.80	1.48	1.62	1.29	1.73	1.73	2.07	1.79
n (G/NG)^d^	66	139	66	139	66	139	66	139	66	139
Correct (%)	80	91	74	95	74	92	74	86	74	86
Fail safe (%)	17	9	26	4	24	4	26	14	26	14
Fail dangerous (%)	3	0	0	1	2	4	0	0	0	0

a Challenge test data from present study (Table 1 and Supplementary Table S1).

b Literature data (Tables 3, 5).

c Number of *μ_max_* values used for calculation of B_f_/A_f_.

d Number of Growth/No-growth responses evaluated.

**Table 7:** Evaluation of the new and existing models for psychrotolerant *B. cereus* by challenge tests and literature data.

Model	Maktabdar et al. (2025; Part 1) with *μ_opt-cal_* of 2.67 h^−1^		Carlin et al. (2013)		Le Marc et al. (2021a)		Le Marc et al. (2024)		Zwietering et al. (1996)	
Environmental factors included in model	Temp, pH, a_w_, acetic, benzoic, citric, lactic, sorbic acids, ortho-, di-, and triphosphates		*panC* group VI strain ADRIA 121; Temp, pH, a_w_		*panC* group V strain B600; Temp, pH		*panC* group II strain MJG03; Temp, pH, a_w_, acetic, lactic		Temp, pH, a_w_	
Datasets	CT^a^	LD^b^	CT	LD	CT	LD	CT	LD	CT	LD^e^
n^c^	41	88	51	82	51	55	–	–	52	66
B_f_	1.07	0.72	1.24	1.09	1.26	1.29	–	–	1.39	1.08
A_f_	1.38	2.58	2.07	1.95	1.78	1.60	–	–	1.54	1.71
n (G-NG)^d^	57	109	67	109	67	105	67	109	67	72
Correct (%)	84	93	78	86	78	72	84	92	78	76
Fail safe (%)	14	7	21	11	21	1	16	8	22	24
Fail dangerous (%)	2	0	1	3	1	27	0	0	0	0

a Challenge test data from present study (Table 1 and Supplementary Table S1).

b Literature data (Tables 4, 5).

c Number of *μ_max_* values used for calculation of B_f_/A_f_.

d Number of Growth/No-growth responses evaluated.

e Data generated by Zwietering et al. (1996) to develop this model was excluded from this model evaluation.

The model developed by Ellouze et al. (2021) predicts simultaneous growth and cereulide formation by an emetic *B. cereus* strain in different matrices. The *μ_opt_* value of 1.78 h^−1^ reported by Ellouze et al. (2021) for dairy-based food was used for evaluation of this model. Ölmez and Aran (2005) developed a reduced polynomial model describing the impact of temperature, pH, NaCl, and lactic acid on growth responses of mesophilic *B. cereus*. Carlin et al. (2013) developed four models for individual mesophilic *B. cereus* strains belonging to *panC* group III and IV. Le Marc et al. (2021a) proposed five models for mesophilic *B. cereus* strains (two strains from *panC* group III and three strains from group IV). Carlin et al. (2013) reported six growth models for individual psychrotolerant *B. cereus* strains from *panC* groups II, V, and VI. Le Marc et al. (2021a) suggested growth models for two psychrotolerant *B. cereus* strains (from *panC* group II and V) by incorporating the effect of temperature on *pH_min_*. Recently, Le Marc et al. (2024) proposed growth boundary models for three psychrotolerant *B. cereus* strains (from *panC* groups II and VI). In contrast to the aforementioned models which were developed based on growth of vegetative cells inoculated into BHI broth with or without added yeast extract and glucose, the study by Zwietering et al. (1996) developed a model specifically for predicting growth of *B. cereus* spores naturally present in milk.

### 2.9 Comparison of the new and extensive models with other available models

The performance of the new mesophilic model (Maktabdar et al., 2025; Part 1) and existing growth models for vegetative mesophilic *B. cereus* (Ölmez and Aran, 2005; Carlin et al., 2013; Ellouze et al., 2021; Le Marc et al., 2021a) were compared by using indices of model performance as indicated in section 2.7. These indices were calculated for predicted and observed growth responses in dairy products. For both the Carlin et al. (2013) and Le Marc et al. (2021a) models, the performances of all suggested mesophilic models for different individual strains were evaluated. The best performing model for predicting growth of both the new challenge tests (See section 2.2) and literature data (See section 2.6) was identified based on B_f_/A_f_ values and on the percentage of correct, fail-safe, and fail-dangerous predictions (section 2.7). In the same way performance of the new psychrotolerant model (Maktabdar et al., 2025; Part 1) and existing psychrotolerant models (Zwietering et al., 1996; Carlin et al., 2013; Le Marc et al., 2021a, 2024) was compared. For the studies of Carlin et al. (2013), Le Marc et al. (2021a), and Le Marc et al. (2024), all proposed psychrotolerant models for individual strains were evaluated and the best performing model was selected.

### 2.10 Data analysis

Challenge test kinetics were fitted using Python 3.11. Data were imported from MS Excel files using the Pandas package (McKinney, 2010; The Pandas Development Team, 2020). The lmfit package was used for curve fitting (Newville et al., 2014). F-test analysis was carried out using the scipy.stats package (Virtanen et al., 2020) to determine the significance of lag time during growth.

## 3 Results

### 3.1 Performance of new mesophilic and psychrotolerant models

The new growth and growth boundary models evaluated in the present study were developed from growth responses in BHI broth and in a dairy solution prepared with 1.45% UF permeate powder (Maktabdar et al., 2025; Part 1). Challenge tests with this and other solutions from dairy ingredients were performed as part of the present study (Table 1, Table 2). The results are briefly summarized here to better understand the potential of the new models studied. Growth rates of Mix-Bc_mes_ or Mix-Bc_psy_ in the 1.45% UF permeate at various temperatures (Table 1 and Supplementary Table S1) were not significantly different (*p* > 0.05) compared to BHI broth experiments performed using Bioscreen C at similar temperatures (Maktabdar et al., 2025; Part 1). Comparable *μ_max_* values were observed in both concentrated and diluted solutions of dairy ingredients under similar environmental conditions (UF permeate 1 and 3; WFC solutions 1, 2, and 3; Supplementary Table S1). Specifically, *μ_max_* values for Mix-Bc_mes_ or Mix-Bc_psy_ determined from concentrated and diluted solutions of (i) UF permeate solutions with 13.9% (concentrated) and 1.38% (×10 diluted) dry matter or for (ii) WFC with 19.1% (concentrated), 9.54% (×2 diluted) and 1.91% (×10 diluted) dry matter did not differ significantly (*p* > 0.05) at 12.5–13.1°C, pH 6.1–6.7 and with <0.02–0.42% water phase salt (WPS; Supplementary Table S1).

To evaluate the performance of the new growth and growth boundary models, the studied matrices included milk, reconstituted infant formula, fermented milk and yogurt, different types of cheeses, desserts, and solutions of dairy ingredients (Tables 1–5). A wide range of product characteristics and storage temperatures were evaluated. The *μ_max_* values in these studies varied from 0.00 to 2.15 h^−1^ for Mix-Bc_mes_, Mix-Bc_psy_, vegetative cells or spores of 50 different known *B. cereus* isolates, as well as naturally occurring *B. cereus* in dairy matrices. The duration of the new challenge tests and of challenge tests from literature was 1–49 days for mesophilic *B. cereus* and 4–90 days for psychrotolerant *B. cereus* (Supplementary Tables S1, S2).

**Figure 1:**
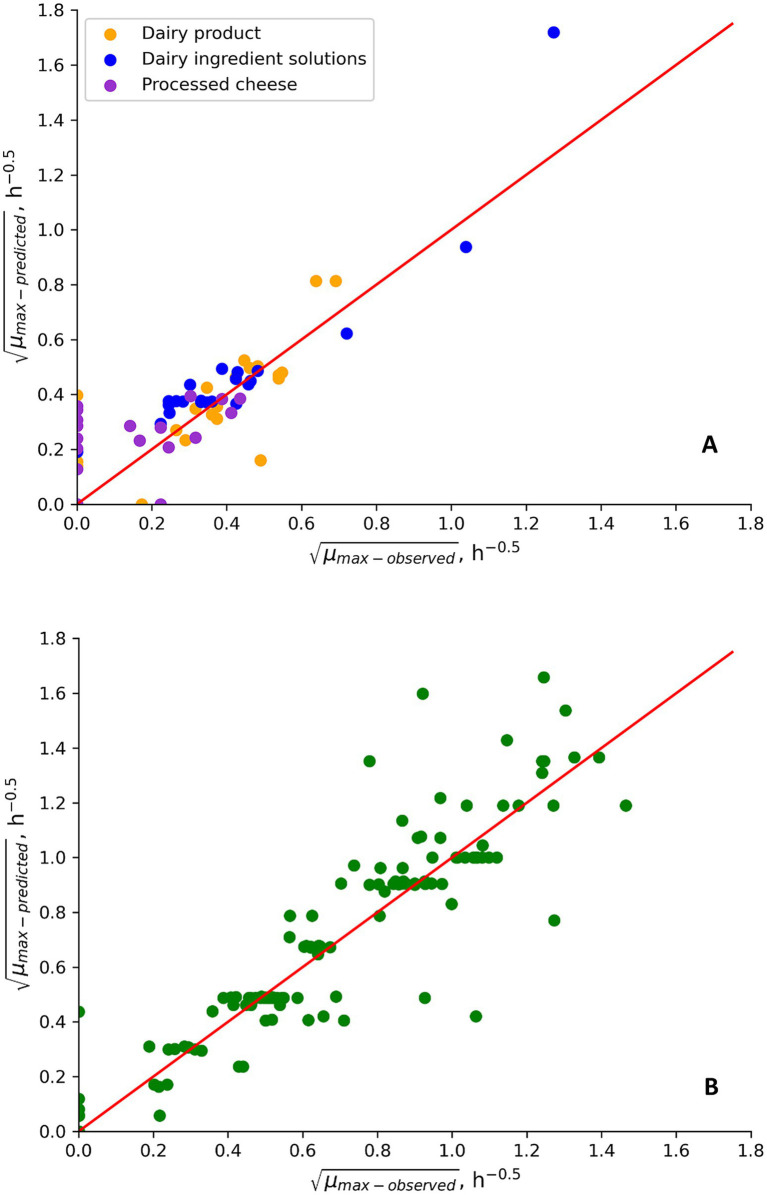
Observed and predicted *μ_max_*-values for the growth behavior of mesophilic *B. cereus* in new challenge tests performed in this study **(A)** and in challenge test data from literature **(B)**. Data points placed on, respectively, the x- and y-axis correspond to fail-dangerous and fail-safe predictions. Other data points correspond to the percentage of correct predictions (Table 6).

The new mesophilic model, without product-calibration of its *μ_opt_* value, showed good performance (B_f_ values of 1.13 and 0.97) in predicting growth rates for both new challenges tests with well characterized dairy matrices and challenge tests from literature where less information on product characteristics was available (Table 6; Figure 1). For the well characterized dairy matrices and new challenges tests, B_f_ values of 0.90 (*n* = 16); 1.20 (*n* = 8) and 1.33 (*n* = 21) were determined for, respectively, different dairy foods, processed cheese, and solutions of dairy ingredients (Results not shown). Specifically for naturally contaminated products the B_f_/A_f_ values were 1.20/1.22 (*n* = 3) for control treatments of new challenge tests and 1.09/1.36 (*n* = 6) for literature data. Due to this performance, it was decided not to carry out any product-calibration of the *μ_opt_* value (2.99 h^−1^) in this model. The prediction of growth/no-growth responses was acceptable (Table 6). Fail-safe predictions resulted primarily from processed cheese at 15–18°C in the new challenge tests and from milk and reconstituted infant formula at 8°C in data from literature (Table 6 and Supplementary Table S1, S2). The two fail-dangerous predictions (3%; Table 6) had *ψ* value of 1.1 indicating that, these growth responses for cream cheese and processed cheese (PC 15) at 15°C, were close to the growth boundary (Supplementary Table S1). By removing the effect of CM terms in the model, one factor at a time, these fail-dangerous predictions were shown to be due to lactic and acetic acid in cream cheese and due to acetic acid in processed cheese.

The psychrotolerant model with a *μ_opt_* value of 2.12 h^−1^ underestimated growth in all 10 challenge tests used for model calibration (Table 2), resulting in B_f_/A_f_ of 0.79/1.37. Product-calibration of the model using this B_f_ value of 0.79 resulted in a *μ_opt-cal_* value of 2.67 h^−1^. The calibrated psychrotolerant model demonstrated a good performance for prediction of 41 growth rates from the new challenge tests with B_f_ of 1.07 and A_f_ of 1.38 (Table 7). B_f_ values of 0.83 (*n* = 15); 1.08 (*n* = 8) and 1.31 (*n* = 18) were determined for, respectively, different dairy foods, processed cheese, and solutions of dairy ingredients (Results not shown). The prediction of growth/no-growth responses (*n* = 57) was acceptable (Table 7). Fail-safe predictions were primarily observed for processed cheese (Supplementary Table S1). One fail-dangerous prediction (2%) was found for cream cheese at 15°C (Table 1 and Supplementary Table S1) and this product had a *ψ*-value of 1.6. As above, by removing the effect of CM terms in the model one factor at a time, the fail-dangerous prediction was shown to be due to lactic and acetic acid in the cream cheese. For dairy matrices and data collected from literature (*n* = 88), the psychrotolerant model underestimated growth rates and its performance was not acceptable with B_f_ of 0.72 and A_f_ of 2.58 (Table 7). At <10°C, the observed growth rates were markedly higher than predicted.

### 3.2 Comparison of the new mesophilic model and available models

The models developed by Carlin et al. (2013) for the *panC* group IV strain F4430/70 (*T_min_* = 9.10°C), and by Le Marc et al. (2021a) for the *panC* group III strain B648 (*T_min_* = 7.39°C), were identified as the best performing models among those proposed for individual strains in these two studies. The new and extensive growth model for Mix-Bc_mes_ had better performed than available mesophilic models in predicting growth responses in various dairy matrices, as determined in the present study (Table 6). Available models had acceptable performance for growth rates in the new challenge tests with Mix-BC_mes_, but the percentages of fail-safe predictions (24–26%) were unacceptably high (Table 6). With data from literature, the performance of the available models, except the model by Ellouze et al. (2021), was good or acceptable, with B_f_ values of 1.10–1.30 and high percentages of correct predictions of 86–95% (Table 6).

### 3.3 Comparison of the new psychrotolerant model and available models

The models developed by Carlin et al. (2013) for *panC* group VI strain ADRIA 121(*T_min_* = 3.30°C), and by Le Marc et al. (2021a) for the *panC* group V strain B600 (*T_min_* = 5.29°C), were identified as the best performing models among the models proposed for different psychrotolrant strains in those studies. For challenge tests in the present study, the performance of the new calibrated model for psychrotolerant *B. cereus* was better than available models, with B_f_ and A_f_ values closer to 1.0 and higher percentages of correct predictions (Table 7). For literature data, the new model predicted growth rates unacceptably and with lower performance than available models (Table 7). For literature data, the growth rate model by Zwietering et al. (1996), with a *T_min_* value of 0°C, performed better than other evaluated models with B_f_ of 1.08 and A_f_ of 1.71. The growth boundary model by Le Marc et al. (2024) which includes the effects of five environmental factors had high percentage of correct predictions for both new challenge tests (84%) and literature data (92%) (Table 7).

### 3.4 Relative lag time (*RLT*) and maximum population density (*N_max_*)

The average *RLT* value for growth of vegetative cells (Mix-BC_mes_) in the new challenge tests was 6.1 ± 6.7 (avg ± SD), indicating considerable variability (*n* = 49; Supplementary Table S1). The corresponding *RLT* value for growth curves of mesophilic *B. cereus* reported in literature for vegetative cells, spores, or naturally occurring cells or spores (*n* = 106) was similar (6.4 ± 3.7; Supplementary Table S2). *RLT* values for both challenge tests with Mix-Bc_mes_ and mesophilic literature data appeared to be independent of storage temperature (*p* > 0.05). The average *RLT* value for growth of vegetative psychrotolerant cells (Mix-BC_psy_) in new challenge tests, was 4.2 ± 6.2 (*n* = 52, Supplementary Table S1). The *RLT* value for Mix-BC_psy_ seemed constant and independent of the storage temperature (*p* = 0.48). In contrast, growth of psychrotolerant cells and/or spores of *B. cereus* reported in literature (Supplementary Table S2) had *RLT* values that increased at lower temperatures (*p* < 0.05), corresponding to *RLT* = (3.5 ± 1.9) + (546 ± 102)/T^2^, where T is temperature in °C. This equation suggests average *RLT* values of 25.3 at 5°C, 9.0 at 10°C, 4.4 at 25°C, and of 3.9 at 37°C.

The log(*N_max_*) of Mix-BC_mes_ in new challenge tests decreased significantly with decreasing storage temperature (*p* < 0.05). The parameters b1 and b2 in Equation 3 had values of 7.67 ± 0.40 and 200 ± 83, respectively. In contrast, log(*N_max_*) for Mix-Bc_psy_ seemed constant, independent of storage temperature (*p* = 0.95), with an average value of 7.15 ± 0.69 log CFU/g.

## 4 Discussion

### 4.1 Validation of new growth models for mesophilic and psychrotolerant *B. cereus*

The present study developed a comprehensive dataset for growth responses of mesophilic and psychrotolerant *B. cereus* (Tables 1–5 and Supplementary Table S1, S2), which enabled a thorough evaluation of the two new and extensive growth and growth boundary models developed by Maktabdar et al. (2025; Part 1) for application in dairy products and solutions (Tables 6, 7). Growth responses from both new challenge tests and literature data allowed for the evaluation of the effect of different types of dairy foods and of their characteristics on the growth responses of *B. cereus* strain cocktails and many different *B. cereus* isolates (Tables 1–5).

**Figure 2:**
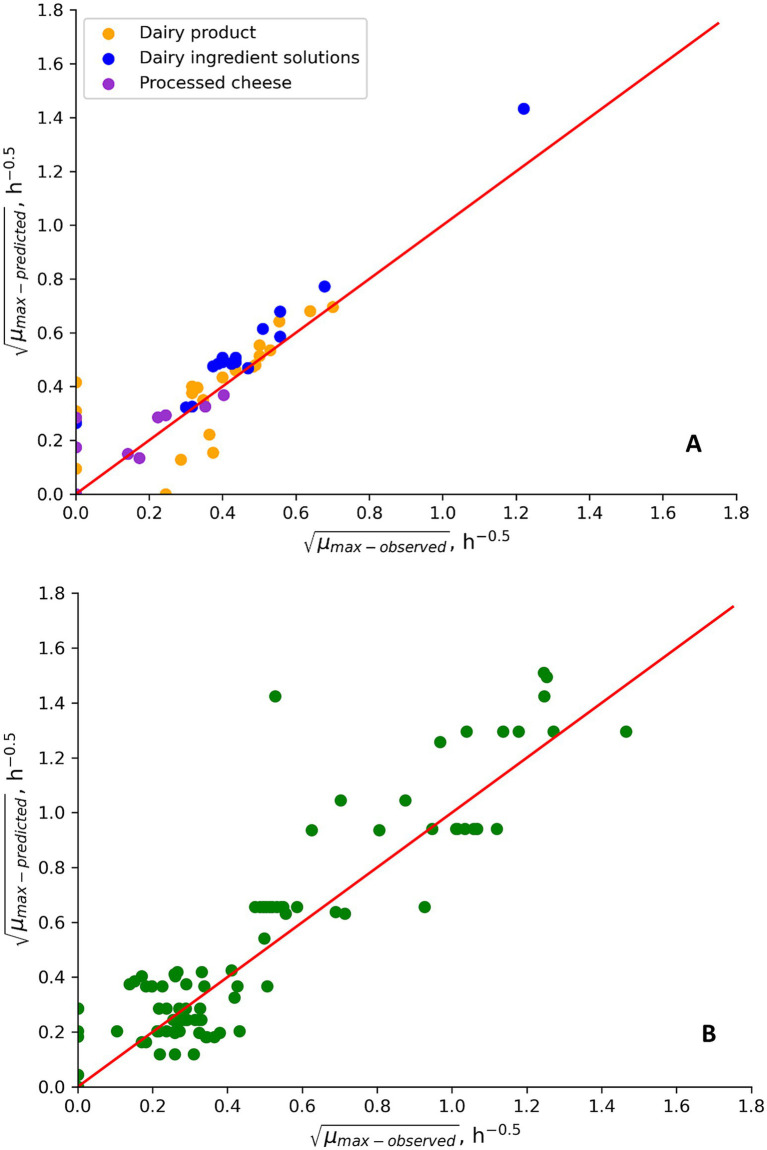
*μ_max_* values predicted by the updated psychrotolerant *B. cereus* model (Maktabdar et al., 2025, Part 1; Figure 3; Equation 7) and observed for growth of psychrotolerant *B. cereus* in new challenge tests performed in this study **(A)** and in challenge tests from literature **(B)**. Data points placed on, respectively, the x- and y-axis correspond to fail-dangerous and fail-safe predictions. Other data points correspond to the percentage of correct predictions.

**Figure 3:**
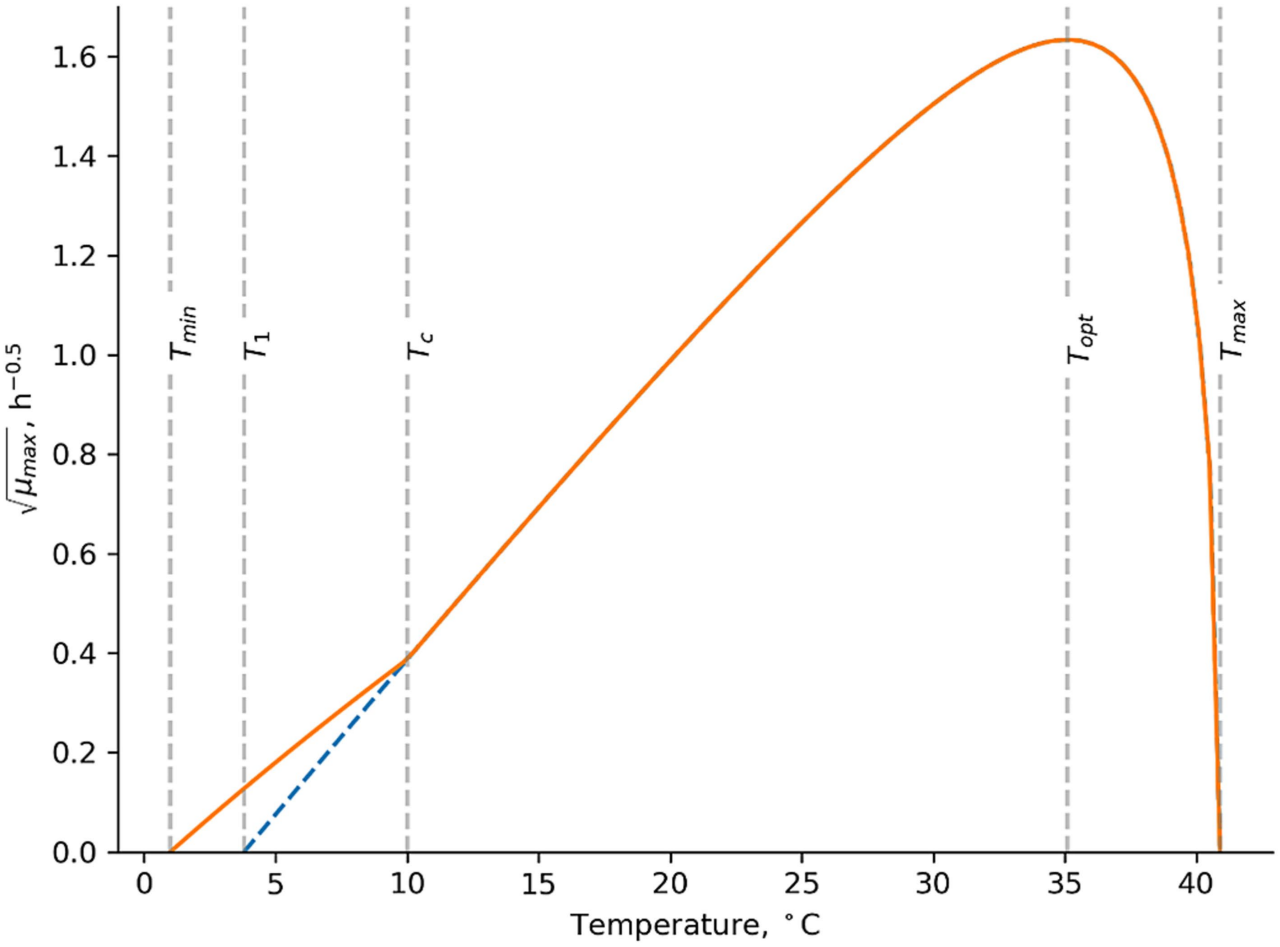
Temperature term suggested by Le Marc et al. (2002). In this example the cardinal parameter values for Mix-Bc_psy_ are used with *T_1_* = 3.80°C, *T_opt_* = 35.1°C and *T_max_* = 40.9°C as determined by Maktabdar et al. (2025; Part 1). *T_min_* = 1.00°C and *T_C_* = 10.0°C were determined in the present study to describe growth rates of psychrotolerant *B. cereus* including *panC* group VI isolates in dairy matrices.

For new challenge tests with well characterized product properties (Table 1 and Supplementary Table S1), the good or acceptable performance of the models (Tables 6, 7; Figures 1A, 2A) showed that both the mesophilic and psychrotolerant models can predict growth responses for a broad range of dairy products. The mesophilic model was developed using a cocktail of *panC* group III isolates (Maktabdar et al., 2025; Part 1). Its good performance for literature data showed that predicted growth responses corresponded to those of many different *B. cereus* strains, including spores in naturally contaminated products (Tables 3, 6; Supplementary Table S2; Figure 1B). A cocktail of dairy isolates (Mix-Bc_psy_) from *panC* group II, III, VI and VIII were used for development of the psychrotolerant model (Maktabdar et al., 2025; Part 1). The unacceptable performance of this model for literature data (B_f_ of 0.72 and A_f_ of 2.58; Table 7) was primarily due to underprediction of growth rates at temperatures below 10°C. These underpredictions were observed for some studies with skimmed and semi-skimmed milk, which were naturally contaminated or inoculated with *B. cereus panC* group VI isolates (Supplementary Table S2; Valík et al., 2003; Ivy et al., 2012; Buehler et al., 2018). The unacceptable performance of the psychrotolerant model at low temperatures suggests that the *panC* group VI isolate (MRB-6) included in the Mix-Bc_psy_ strain cocktail has a higher *T_min_* value than some *B. cereus* isolates naturally occurring in milk. To overcome this limitation of the Maktabdar et al. (2025; Part 1) psychrotolerant model the temperature term in that model has been replaced by the temperature term suggested by Le Marc et al. (2002) for growth of *L. monocytogenes* (Figure 3; Equation 7)


(7) $$\begin{array}{l} CM\left(T\right)=\\ \left\{ \begin{array}{cc} \frac{\left(T-T_{\text{max}}\right){\left(T-T_{1}\right)}^{n}}{\left(T_{\mathrm{opt}}-T_{1}\right)\left[\begin{array}{l} \left(T_{\mathrm{opt}}-T_{1}\right)\left(T-T_{\mathrm{opt}}\right)\\ -\left(T_{\mathrm{opt}}-T_{\text{max}}\right)\left(\left(n-1\right)T_{\mathrm{opt}}+T_{1}-nT\right) \end{array}\right]} & T\geq T_{C}\\ \frac{\left(T_{c}-T_{\text{max}}\right){\left(T_{c}-T_{1}\right)}^{n}}{\left(T_{\mathrm{opt}}-T_{1}\right)\left[\begin{array}{l} \left(T_{\mathrm{opt}}-T_{1}\right)\left(T_{c}-T_{\mathrm{opt}}\right)\\ -\left(T_{\mathrm{opt}}-T_{\text{max}}\right)\left(\left(n-1\right)T_{\mathrm{opt}}+T_{1}-nT_{c}\right) \end{array}\right]}{\left(\frac{T-T_{\text{min}}}{T_{C}-T_{\text{min}}}\right)}^{2} & T_{\text{min}}<T<T_{C} \end{array}\right. \end{array}$$

where *T* is the temperature (°C), *T_c_* is the change temperature; *T_1_* is the x-intercept of the slope; *T_min_* is the theoretical minimum growth temperature (°C); and *T_opt_* and *T_max_* are the optimum and maximum growth temperatures (°C), respectively.

Replacing the temperature term in the Maktabdar et al. (2025; Part 1) psychrotolerant model with Equation 7 and using a *T_min_* value of 1.00°C and a *T_C_* value of 10.0°C improved the previously unacceptable B_f_/A_f_ values of 0.72/2.58 in Table 7 to become 1.07/1.80 (*n* = 88). The corresponding percentages of correct, fail safe and fail dangerous predictions were 91, 9 and 0% (Figure 2B). For the new challenge tests from the present study the updated model resulted in B_f_/A_f_ values of 1.07/1.39 (*n* = 41) and the corresponding percentages of correct, fail safe and fail dangerous predictions were 82, 16 and 2% (*n* = 57) (Figure 2A). Specifically for naturally contaminated products the B_f_/A_f_ values were 1.09/1.12 (*n* = 3) for control treatments of new challenge tests and 0.79/1.40 (*n* = 30) for literature data (Results not shown). The markedly improved performance of the updated psychrotolerant models was obtained by using Equation 7 and by keeping *T_1_*, *T_opt_* and *T_max_* values as determined for Mix-Bc_psy_ by Maktabdar et al. (2025; Part 1) (Figure 3). The performance of the updated psychrotolerant model showed that predicted growth responses corresponded to those of many different *B. cereus* strains, including spores in naturally contaminated products (Tables 4, 5; Supplementary Table S2; Figure 2B). The approach used here to expand the psychrotolerant model by Maktabdar et al. (2025; Part 1) for predicting growth of *B. cereus* in naturally contaminated dairy products at low temperatures (Figure 3; Equation 7) may also be applicable for other environmental conditions, if relevant data become available. As one example Sarkar et al. (2023) observed growth at 50°C for a mesophilic *B. cereus* strains cocktail in paneer. If other studies confirm growth of mesophilic *B. cereus* in dairy products at temperatures above *T_max_* of the new mesophilic model by Maktabdar et al. (2025; Part 1) (45.4°C) then the temperature term of that model may be updated. This can be obtained by using Equation 7 but introducing the modification of the model at high instead of at low temperatures (Equation 7; Figure 3).

Importantly, the performed model evaluation studies indicated the suitability of the Mix-Bc_mes_ and Mix-Bc_psy_ strain-cocktails proposed by Maktabdar et al. (2024) for challenge tests with a variety of dairy products (Table 6; Figure 2). However, the Mix-Bc_psy_ strain-cocktail is not optimal for challenge tests at temperatures below 8–10°C. For challenge tests at these low temperatures, the Mix-Bc_psy_ strain-cocktail should be supplemented with at least one *B. cereus panC* group VI isolates with a *T_min_* value of close to 1.0°C. Alternatively, the updated psychrotolerant model, evaluated in the present study, can be used to predict growth responses of *B. cereus* at temperatures down to 4°C.

For the studied 371 growth responses, a total of three fail-dangerous predictions (0.8%) with *ψ* values ranging from 1.1 to 1.6 were observed for both the mesophilic and the psychrotolerant models (Tabled 6, 7; Section 3.1). This performance was considered acceptable, as a small percentage of fail-dangerous predictions (below 5%) can occur, even with a precise model due to variability in product characteristics and storage conditions close to the growth boundary (*ψ* value = 1.0) (Mejlholm and Dalgaard, 2009; Mejlholm et al., 2010). To efficiently prevent growth of *B. cereus*, environmental conditions corresponding to a *ψ* value of 2.0 or above can be used, as previously suggested for *L. monocytogenes* and *C. botulinum* (Mejlholm et al., 2010; Koukou et al., 2021, 2022b).

The range of applicability (RoA) of the new extensive models was identified from the conditions within which their performance was acceptable as previously suggested and applied for other microorganisms (Dalgaard and Mejlholm, 2019; Martinez-Rios et al., 2019; Koukou et al., 2022b). The RoA for the mesophilic model included temperatures (10°C–45°C), pH (4.8–7.3), WPS (<6.6%, corresponding to calculated a_w_ > 0.960), water phase concentrations of organic acids (acetic acid <1,534 ppm; benzoic acid <362 ppm; citric acid <8,126 ppm; lactic acid <17,472 ppm; sorbic acid <155 ppm) and water phase concentrations of phosphate melting salts (orthophosphate <3.1%; diphosphate <1.7%; triphosphate <2.1%). For the updated psychrotolerant model the RoA included temperatures (4°C–37°C), pH (4.7–7.3), WPS (< 5.1%, corresponding to calculated a_w_ > 0.970), water phase concentrations of organic acids (acetic acid <1,534 ppm; benzoic acid <155 ppm; citric acid <8,126 ppm; lactic acid <17,472 ppm; sorbic acid <103 ppm), and water phase concentrations of phosphate melting salts (orthophosphate <3.1%, diphosphate <1.7%, and triphosphate <1.7%).

### 4.2 Application of new growth models for mesophilic and psychrotolerant *B. cereus*

The two new and validated models can be used to predict the combined effect of many relevant product characteristics and storage temperatures on growth and growth boundary of mesophilic and psychrotolerant *B. cereus* in various dairy products and dairy ingredient solutions (Tables 6, 7; Section 4.1). Previous dairy product validation studies primarily showed that other predictive models were applicable to milk, infant formula, and paneer at different storage temperatures (Zwietering et al., 1996; Valík et al., 2003; Buss da Silva et al., 2017; Sarkar et al., 2023). The new, updated and validated models are therefore important to support the evaluation and management of growth for *B. cereus* subgroups in a range of dairy foods.

The lag phase for spores and vegetative cells of *B. cereus* can be important when evaluating the time required to reach critical concentrations. The *RLT* approach has previously been used to predict lag time for *B. cereus* and several other microorganisms (Ross and Dalgaard, 2004; Ölmez and Aran, 2005). The comprehensive dataset analyzed in the present study showed considerable variability in *RLT* values for growth of *B. cereus. RLT* values varied within 0–29 in the new challenge tests with vegetative cells and within 0–12 or 0–83, respectively, for mesophilic or psychrotolerant vegetative cells and/or spores from literature data (Tables 1–5). Significant variation in *RLT* values for *B. cereus* is not surprising, as heat treatment during food processing and many other factors can influence germination and outgrowth of *B. cereus* subgroups (Augustin, 2011; Koseki and Nonaka, 2012). Due to the documented variability of *RLT* values for *B. cereus* in dairy matrices, it is recommended to use the new and extensive growth and growth boundary models either without lag time or using relatively low average *RLT* values. Specifically, *RLT* values of 6.1 for the mesophilic model and 4.2 for the psychrotolerant model, as estimated for new challenge tests in the present study, are recommended.

Unpasteurized dairy products are likely to contain relatively more psychrotolerant *B. cereus s.l.* subgroups compared to heated products. However, both mesophilic and psychrotolerant *B. cereus s.l.* subgroups can be present in a wide range of heated dairy matrices (Carlin et al., 2010; Maktabdar et al., 2024). The high prevalence and concentrations of up to approximately 100 CFU/g or CFU/mL in milk and dairy powders highlights the importance of managing *B. cereus s.l.* in dairy foods (Tirloni et al., 2022; Maktabdar et al., 2024). Options include (i) inactivation, for example by ultra-high-temperature treatment, or (ii) reducing or preventing growth, for example by chilling or by proper product formulations (EFSA, 2005). It can therefore be interesting to use predictions from both the new mesophilic and psychrotolerant *B. cereus* models simultaneously to support evaluation and management of their growth. Chilled cottage cheese is used here as an example of how the new models can be used. This product can have 75% moisture, 4% lipid, pH 5.2, 1.1% WPS, 1,250 ppm water phase lactic acid, and may contain sorbic acid as a food additive (Østergaard et al., 2014). At 8°C, the predicted time for growth of psychrotolerant *B. cereus* from 10 CFU/g to a critical concentration 1,000 CFU/g was 11 days without a lag phase, and 18 days when including a lag time corresponding to a *RLT* value of 4.2. Raising the temperature to 10°C, reduced the predicted time to critical growth from 18 to 8 days. This growth time, however, could be extended to 25 days by either reducing the product pH from 5.2 to 5.0 or by adding 54 ppm water phase sorbic acid. For this cottage cheese with pH 5.2 and at 10°C, mesophilic *B. cereus* is predicted to grow from 10 CFU/g to 1,000 CFU/g in 16 days without lag time, and in 31 days when including a lag time corresponding to a *RLT* value of 6.1. These predictions indicate that psychrotolerant *B. cereus* are more critical than mesophilic *B. cereus* for the safe shelf-life of cottage cheese stored at 8–10°C.

To facilitate correct application of the two extensive growth and growth boundary models for *B. cereus*, they will be integrated into the user-friendly Food Spoilage and Safety Predictor (FSSP) software^2^ to be applied within their defined RoA and with recommended lag times. This integration will guide users in providing model inputs, including pH, a_w_, and organic acid concentrations, that aligns with the developments and recommended usage of these models. The two models were developed using direct pH measurements for solid dairy samples (See section 2.5). Therefore, pH must be measured in the same ways for products where predictions are needed. This is important, as pH of processed cheese, for instance, can be about 0.4 units lower when measured by a direct probe compared to a 5- or 10-fold diluted sample. Regarding a_w_, the two models were developed using liquid matrices where a_w_ was calculated from the concentration of NaCl in the water phase (Maktabdar et al., 2025; Part 1). Therefore, when applying the models, water phase salt must be used as input to obtain un-biased predictions, rather than the measured a_w_ of samples. To illustrate the quantitative effect of this aspect, the mesophilic model provided predictions for processed cheese in new challenge tests with B_f_ of 1.12, corresponding to 12% overestimation of growth rates. However, when measured a_w_ values were incorrectly used as model input, the B_f_ decreases to 0.59, corresponding to 41% underestimation of growth rates (Results not shown).

Numerous predictive models for various microbial species, including *B. cereus*, have been developed at different constant temperatures and subsequently successfully applied to predict growth at dynamic temperature conditions in food (Juneja et al., 2019; Østergaard et al., 2014; Martinez-Rios et al., 2016; Park et al., 2021). However, we have found no studies evaluating *B. cereus* growth models for dairy products under dynamic temperatures storage conditions. In the present study, the two new and extensive *B. cereus* growth models were validated at constant storage temperatures and for constant product characteristics determined or estimated at the start of the storage period (Tables 1, 3, 4). We expect these models to be applicable for dynamic temperatures and product characteristics, as observed for other pathogens and foods. However, further research is required to evaluate the performance of *B. cereus* growth models under dynamic conditions. For some dairy foods, including different types of cheeses, lactic acid consumption during storage and distribution can lead to an increase in product pH, creating more favorable conditions for microbial growth (Martinez-Rios et al., 2020; Mihaly Cozmuta et al., 2020; Song et al., 2022). When using the two new *B. cereus* growth models it is recommended to obtain information on changes in dairy product pH and lactic acid concentration during storage. Alternatively, the models should not be applied for storage times exceeding those in challenge tests used for model validation, which extend up to 49 days for the mesophilic model and up to 90 days for the psychrotolerant model (Supplementary Tables S1, S2).

The new mesophilic and psychrotolerant *B. cereus* models are more extensive than available models (Tables 6, 7). Nevertheless, future studies could expand these models by incorporating terms for growth-inhibiting effects of storage atmosphere, including CO_2_ and O_2_ (Sutherland et al., 1996; Couvert et al., 2023), nisin (Martinez-Rios et al., 2021), or the simultaneous growth of lactic acid bacteria (Røssland, 2003; Østergaard et al., 2014). Furthermore, it would be interesting to evaluate the performance of these models for other food categories.

### 4.3 Evaluation and comparison of performance for new and available models

Of the eight studied available models, only predictions by Zwietering et al. (1996) and Ölmez and Aran (2005) models have been previously compared with *B. cereus* growth responses in dairy products, specifically in milk, reconstituted milk, and infant formula. The present product validation study expanded the range of dairy foods and dairy solutions used to evaluate the performance of all the studied models (Tables 1–5). For growth responses in new challenge tests (Tables 1, 2), higher B_f_ values (five of seven models), higher A_f_ values, and higher percentages of fail-safe predictions (seven of eight models) were observed for available models compared to the new models (Tables 6, 7). This was primarily because available models did not account for the growth-inhibiting effects of acetic, benzoic, citric, and sorbic acids, as well as for phosphate melting salts which were present in products like processed cheese. Similar observations have been previously reported with other incomplete models for different pathogens and foods (Mejlholm et al., 2010; Martinez-Rios et al., 2019; Koukou et al., 2021, 2022b). However, strain variability may also have contributed to these observed effects, as available models were developed using strains other than those included in the new challenge tests. The performed evaluation and comparison of new and available models with data from new and literature challenge tests suggest that differences between observed and predicted growth responses were influenced by variability in product characteristics, and with strain variability being primarily important at low storage temperatures (Tables 6, 7). The model by Ellouze et al. (2021) did not follow this pattern as it exclusively considered the effect of temperature.

Differences in the performance of the new and the available models were further evaluated by comparing selected growth boundary predictions. For the processed cheese as discussed by Maktabdar et al. (2025; Part 1) (25°C; 55% moisture, 25% lipid, 2.0% WPS, 2.0% water phase orthophosphate and 1,500 ppm, 4,500 ppm and 14,000 ppm in water phase of acetic, citric and lactic acids, respectively), the new mesophilic and psychrotolerant models predicted pH-growth-boundaries (*μ_max_* = 0.00; *Ψ* = 1.00) at pH of 5.65 and of 5.73, respectively. The corresponding pH-growth-boundaries of the available models, which included the effect of pH but did not account for interaction between environmental factors, varied from pH 1.9 (Ölmez and Aran, 2005) to 4.96 (Carlin et al., 2013). Clearly, growth boundary predictions differed markedly between these available models and the new and extensive models. These differences were due to the higher number of environmental factors included in the new models and, importantly, the effect of their interactions, as modelled by Le Marc et al. (2002) approach (Maktabdar et al., 2025; Part 1). The studied available models by Le Marc et al. (2021a) and by Le Marc et al. (2024) included the effect of interaction between environmental factors. However, for the processed cheese discussed above (pH 5.73), the psychrotolerant model by Le Marc et al. (2021a), predicted rapid growth at 25°C and pH-growth-boundaries of 4.7, 5.7, and 7.2 at 25°C, 10°C and 8°C, respectively. These high pH-growth-boundaries at 10°C and 8°C resulted in fail-dangerous predictions for different dairy products (Table 7). In contrast the psychrotolerant model by Le Marc et al. (2024), which includes the effect of temperature, pH, a_w_, acetic and lactic acids, predicted a pH-growth-boundary of 5.68 for the processed cheese at 25°C, and this was very similar to the prediction of 5.73 by the new psychrotolerant model. These similar predictions of growth boundaries for processed cheese by two different extensive models support the conclusion that extensive models, which include the effect of interaction between their environmental factors, are required to obtain realistic predictions of growth and growth boundaries for complex dairy products, as previously observed for other pathogens and foods (Mejlholm et al., 2010).

### 4.4 Calibration of *μ_opt_* and selection of cardinal parameter values

The product-calibrated specific growth rate at optimum growth temperature (*μ*_*opt*-cal_) of 2.67 h^−1^ for the new psychrotolerant model was higher than the *μ_opt_* value of 2.12 h^−1^ obtained from temperature growth experiments using BHI broth with pH 6.0 (Maktabdar et al., 2025; Part 1). The pH of 6.0 was chosen to use a single pH value when quantifying effects of temperature, NaCl/a_w_, organic acids, and phosphate melting salts during model development (Maktabdar et al., 2025; Part 1). Consequently, the estimated *μ_opt_* value of Mix-Bc_psy_ in BHI broth was lower than it would have been at the optimum pH for growth of 7.14 (Maktabdar et al., 2025; Part 1). Therefore, the higher *μ*_*opt*-cal_ value 2.67 h^−1^ does not indicate that Mix-Bc_psy_ grow faster in dairy products compared to BHI broth.

The two new and extensive *B. cereus* growth models, evaluated in the present study, included cardinal parameters values for pH, lactic and citric acids which were determined from growth in UF permeate (Maktabdar et al., 2025; Part 1). Including these values, rather than exclusively cardinal parameter values determined using BHI broth, was quantitatively important for the ability of these models to provide un-biased predictions of growth responses in dairy matrices (Tables 6, 7; Section 4.1). Maktabdar et al. (2025; Part 1) discussed potential explanations for differences in cardinal parameter values determined using BHI broth and UF permeate. Here the quantitative effects of these differences on predictions are highlighted. The mesophilic model, including only cardinal parameter values determined using BHI broth, predicted growth of Mix-Bc_mes_ in new challenge tests with B_f_/A_f_ of 0.92/1.46 and 5% fail-dangerous predictions, with *ψ*-values ranging from 1.3 to >10 (Result not shown). Using relevant cardinal parameter values from UF permeate and a conservative approach to obtain the widest growth ranges significantly improved these predictions, resulting in B_f_/A_f_ of 1.13/1.49 and 3% fail-dangerous predictions with *ψ*-values <1.1 (Table 6). A similar improvement was also observed for performance of the psychrotolerant model. With cardinal parameters values exclusively from BHI broth the predicted growth in new challenge tests had B_f_/A_f_ of 1.13/1.47 and 5% fail-dangerous predictions with *ψ*-values >10 (Result not shown). Using selected cardinal parameter values from UF permeate improved the B_f_/A_f_ to 1.07/1.38 and reduced the percentage of fail-dangerous predictions to 2% with a *ψ*-values of 1.6 (Table 7). This emphasized the importance of using cardinal parameter values from the dairy based UF permeate together with cardinal parameter values from BHI broth. Further studies are needed for a better qualitative and quantitative understanding of the effect of dairy matrices on estimation of cardinal parameter values (Maktabdar et al., 2025; Part 1).

The new mesophilic and psychrotolerant models, modified to include the effect of undissociated citric acid, exhibited 8 and 7% fail-dangerous predictions with *ψ*-values of 8.7 and 3, respectively (Result not shown). However, by incorporating terms for total citric acid, the performance of both models improved, and fail-dangerous predictions specifically related to citric acid were eliminated (Tables 6, 7). Several predictive models previously included the inhibitory effect of total organic acid concentrations (Presser et al., 1997, 1998; Rosso et al., 1997; Ross et al., 2003). However, other successfully validated models included terms for undissociated citric acid to predict growth in dairy products and other foods (Mejlholm and Dalgaard, 2009; Martinez-Rios et al., 2019; Koukou et al., 2021, 2022b). As pointed out by Maktabdar et al. (2025; Part 1), a better understanding of interaction between organic acids and food matrices would facilitate development of un-biased predictive models.

## 5 Conclusion

This study successfully validated two extensive predictive models for mesophilic and psychrotolerant *B. cereus* growth in dairy matrices and determined the models’ RoA. Both models were able to predict *B. cereus* growth responses for different types of dairy matrices, diverse product characteristics, a wide range of storage temperatures, and for foods that were naturally contaminated or inoculated with different *B. cereus* isolates. Predictions from these validated models can contribute to development and re-formulation of dairy products, risk assessment, estimation of safe shelf-life, and thereby enhance food safety through improved management of mesophilic and psychrotolerant *B. cereus* in a broad range of dairy products. We recommend using the models without lag time or using low average *RLT* values of 6.1 for the mesophilic model and 4.2 for the psychrotolerant model. A *ψ* value of 2.0 or above can be used to identify conditions that effectively prevent *B. cereus* growth. To facilitate the correct application of these models, they will be included in the user-friendly Food Spoilage and Safety Predictor (FSSP) software. Further research is needed to evaluate the performance of the new models under dynamic environmental conditions, for other food matrices, and to more accurately predict lag time for *B. cereus* in dairy and other food products.

## Data Availability

The original contributions presented in the study are included in the article/Supplementary material, further inquiries can be directed to the corresponding author.

## Footnotes

^1^ https://automeris.io/

^2^ http://fssp.food.dtu.dk
