# Supplementary material for: Evaluation and validation of extensive growth and growth boundary models for mesophilic and psychrotolerant Bacillus cereus in dairy products (Part 2)
Source: Front Microbiol. 2025 Mar 31;16:1553903. doi: 10.3389/fmicb.2025.1553903 (PMC11994723; doi:10.3389/fmicb.2025.1553903)
Supplement: Supplementary file 3 [file Data_Sheet_1.docx]

# Supplementary material: Description of the predictive models for *Bacillus cereus* growth evaluated in the present study

## S.1. The models by Carlin et al. (2013):

This study developed several cardinal parameter models for growth of individual *B. cereus* strains. These models include the effects of temperature, pH, and water activity (a_w_) (Eq. 1).

|  | $\mu_{max}= \mu_{opt} . CM\left( T \right)\cdot CM\left( pH \right)\cdot CM(a_{w})$ | 1 |
| --- | --- | --- |

where *CM* describes the effect of cardinal model terms and *µ_opt_* is the maximum specific growth rate (h^-1^) at optimum condition. The effect of temperature, pH and a_w_ were described by Eq. 2.

|  | $CM\left( X \right)= \left\{ \begin{matrix} 0 & X\leq X_{min} \\ \frac{(X-X_{max}){(X-X_{min})}^{n}}{(X_{opt}-X_{min})\left[ \left( X_{opt}-X_{min} \right)\left( x-X_{opt} \right)-(X_{opt}-X_{max})((n-1)X_{opt}+X_{min}-nX) \right]} & X_{min}<X<X_{max} \\ 0 & X\geq X_{max} \end{matrix} \right.$ | 2 |
| --- | --- | --- |

where *X_max_*, *X_opt_*, and *X_min_* are maximum, optimum, and minimum temperatures, pH, and a_w_ for growth. The value of n for the effect of temperature was set to 2, and for pH and a_w_ to 1.

Cardinal parameter values for mesophilic and psychrotolerant strains are listed in Table 1.

Table 1. Cardinal parameters for individual strains (Carlin et al., 2013)

|  | *panC* group | *µ_opt_* (h^-1^) | *T_min_* (^o^C) | *T_opt_* (^o^C) | *T_max_* (^o^C) | *pH_min_* | *pH_opt_* | *a_wmin_* |
| --- | --- | --- | --- | --- | --- | --- | --- | --- |
| Mesophilic strains | | | | | | | | |
| F4810/72 | III | 3.12 | 7.9 | 38.7 | 48.0 | 4.63 | 6.68 | 0.941 |
| F837/76 | III | 3.32 | 7.4 | 38.9 | 48.0 | 4.65 | 6.39 | 0.944 |
| F4430/73 | IV | 3.27 | 9.1 | 38.3 | 48.0 | 4.59 | 6.42 | 0.946 |
| ATCC 14579 | IV | 2.76 | 7.8 | 37.4 | 48.0 | 4.60 | 6.73 | 0.956 |
| Psychrotolerant strains | | | | | | | | |
| RIV BC120 | II | 2.62 | 1.4 | 36.8 | 41.0 | 4.68 | 6.51 | 0.946 |
| NVH 0861-00 | II | 2.72 | 5.1 | 35.0 | 40.2 | 4.62 | 6.43 | 0.950 |
| F2769/77 | V | 2.81 | 5.1 | 36.0 | 41.0 | 4.87 | 6.51 | 0.956 |
| NHV 141 | V | 2.82 | 5.2 | 36.2 | 41.0 | 4.69 | 6.45 | 0.949 |
| KBAB4 | VI | 1.83 | 3.9 | 31.0 | 40.9 | 4.85 | 6.49 | 0.964 |
| ADRIA 121 | VI | 2.59 | 3.3 | 36.1 | 41.0 | 4.96 | 6.39 | 0.973 |

## S.2. Model by (Le Marc et al., 2021):

This study developed several cardinal models for individual mesophilic and psychrotlerant strains by combining the effects of temperature and pH (Eq. 3). The effect of temperature and pH was described using Eq. 2 and 4, respectively.

|  | $\mu_{max}= \mu_{opt} . CM\left( T \right)\cdot CM\left( pH \right)$ | 3 |
| --- | --- | --- |

|  | $CM(pH) = 1- {10}^{n({pH}_{min}- pH)}$ | 4 |
| --- | --- | --- |

where n is a shape parameter and set to 0.90. The effect of temperature on *pH_min_* was included in the models using Eq. 5.

|  | $\left\{ \begin{matrix} {pH}_{min}(T)= {pH}_{ref} - ({pH}_{ref} - {pH}_{min0}){(1-{(\frac{T_{mes}-T}{T_{mes}-MINt})}^{\eta_{1}})}^{\frac{1}{\kappa}} & if T\leq T_{mes} \\ {pH}_{min}(T)= {pH}_{ref} - ({pH}_{ref} - {pH}_{min0}){(1-{(\frac{T - T_{mes}}{T_{max}- T_{mes}})}^{\eta_{2}})}^{\frac{1}{\kappa}} & if T>T_{mes} \\ {pH}_{min}(T)={pH}_{ref} & if T \leq MINt \end{matrix} \right.$ | 5 |
| --- | --- | --- |

where *pH_ref_* is reference pH which was set to 7.2, *pH_min0_* is the lowest *pH_min_* assumed to be obtained at *T_opt_*, which was set to 4.7 for all mesophilic and psychrotolerant strains. *T_mes_* is the temperature of maximum enzyme stability. The cardinal parameter values for each strain are listed in Table 2.

Table 2. Cardinal parameters for individual strains (Le Marc et al., 2021)

|  | *panC* group | *µ_opt_* (h^-1^) | *T_min_* (^o^C) | *T_opt_* (^o^C) | *T_max_* (^o^C) | *T_mes_* (^o^C) | *MINt* (°C) |
| --- | --- | --- | --- | --- | --- | --- | --- |
| Mesophilic strains | | | | | | | |
| B401 | IV | 3.30 | 5.91 | 40.07 | 46.3 | 27.1 | 9.78 |
| B632 | IV | 3.55 | 7.31 | 41.32 | 48.66 | 29.2 | 10.77 |
| B648 | III | 3.46 | 7.39 | 41.06 | 46.86 | 28.1 | 10.83 |
| B663 | IV | 3.23 | 5.81 | 39.88 | 47.08 | 27.4 | 9.71 |
| B672 | III | 3.49 | 6.97 | 41.53 | 48.78 | 29.5 | 10.53 |
| Psychrotolerant strains | | | | | | | |
| B639 | II | 2.24 | 3.26 | 34.31 | 39.05 | 21.5 | 7.89 |
| B600 | V | 2.71 | 5.29 | 36.94 | 40.86 | 24.6 | 9.34 |

## S.3. The model by Ellouze et al. (2021):

This study developed a model predicting *B. cereus* growth and cereulide formation. An emetic mesophilic strain (F4810/72) was used for model evaluation. The growth model primarily includes the effect of temperature (Eq. 6). The effect of temperature was included in the model using Eq. 2.

|  | $\mu_{max}= \mu_{opt}\cdot CM\left( T \right)$ | 6 |
| --- | --- | --- |

The value for *µ_opt_* was adjusted to 1.78 (h^-1^) to describe the growth of *B. cereus* in dairy products. The cardinal values for the effect of temperature used for model evaluation are reported in Table 3.

Table 3. Cardinal parameters for the F4810/72 strain (Ellouze et al., 2021)

| *µ_opt_* (h^-1^) | *T_min_* (^o^C) | *T_opt_* (^o^C) | *T_max_* (^o^C) |
| --- | --- | --- | --- |
| 1.78 | 7.99 | 39.66 | 47.84 |

## S.4. The model by Ölmez and Aran (2005):

This study developed a reduced polynomial model for *B. cereus* growth. A cocktail of three mesophilic *B. cereus* strains (emetic DSMZ 4312, diarrheal DSMZ 4313, and NRRL B-3711 strains) were used for model development. The model includes the effect of temperature, pH, a_w_, and lactic acid (Eq. 7).

|  | ln GR=-20.251+0.411*T+3.743*PH-0.0096*NAL-0.0034*T^2^-0.2220*PH^2^+0.00000603*NACL^2^-0.0203*T*PH-0.00015*T*NAL-0.000058*T*NACL+0.0013*PH*NAL-0.00066*PH*NACL | 7 |
| --- | --- | --- |

where GR is growth rate (CFU/g/h), T is temperature (^o^C), PH is the pH, NAL is sodium lactate or total lactic acid concentration (mM), and NACL is the water phase NaCl concentration (mM). The value for *µ_max_* (h^-1^) was calculated by multiplying GR with ln(10).

## S.5. The model by Zwietering et al. (1996):

This study developed a cardinal model for growth of psychrotolerant *B. cereus* in milk by incorporating the effect of temperature, pH, and a_w_ (Eq. 1). The effect of temperature, pH, and a_w_ were described using Eq. 8, 9, and 10, respectively. The cardinal parameter values of the model are reported in Table 4.

|  | $CM(T) = {(\frac{T - T_{min}}{T_{opt} - T_{min}})}^{2}$ | 8 |
| --- | --- | --- |

|  | $CM(pH) = \frac{(pH - {pH}_{min})(2\cdot{pH}_{opt}-{pH}_{min}- pH)}{{({pH}_{opt}-{pH}_{min})}^{2}}$ | 9 |
| --- | --- | --- |

|  | $CM(a_{w}) = \frac{a_{w} - a_{w,min}}{1 - a_{w,min}}$ | 10 |
| --- | --- | --- |

Table 4. Cardinal parameters for psychrotolerant *B. cereus* strain (Zwietering et al., 1996)

| *µ_opt_* (h^-1^) | *T_min_* (^o^C) | *T_opt_* (^o^C) | *pH_min_* | *pH_opt_* | *a_w,min_* |
| --- | --- | --- | --- | --- | --- |
| 2.00 | 0.00 | 37.00 | 4.49 | 6.50 | 0.950 |

## S.6. The model by Le Marc et al. (2024):

This study developed growth boundary models for three individual psychrotolerant *B. cereus* strains. These models evaluated the effect of temperature, pH, a_w_, acetic and lactic acids on the growth/no-growth interface. The effects of temperature and pH were described by Eq. 2 and 4, respectively. Effect of a_w_ was included using Eq. 11.

|  | $CM\left( a_{w} \right)= \left\{ \begin{matrix} 1, & 1<a_{w} \geq a_{w,opt} \\ \frac{{(a}_{w}-1)-{(a}_{w}-a_{w,min})}{{(a}_{w}-a_{w,min}){(a}_{w}-1)-{{{(a}_{w}-a}_{w,opt})}^{2}} & a_{w,min} < a_{w}< a_{wopt} \\ 0, & a_{w} \leq a_{w,min} \end{matrix} \right.$ | 11 |
| --- | --- | --- |

where *a_w,opt_* is the optimum a_w_ which was set to 0.997. Effects of undissociated acetic acid (AACu) and undissociated lactic acid (LACu) were included using Eq. 12.

|  | $CM({{AAC}_{u}, LAC}_{u}) = 1-\left( \frac{\left[ HA \right]}{{MIC}_{u}} \right)^{\alpha}$ | 12 |
| --- | --- | --- |

where [HA] is the concentration of undissociated acetic or lactic acid (mM), MICu is the theoretical minimum inhibitory concentration of undissociated acids (mM), and α is the shape parameter with values as reported in Table 5.

Evaluating growth/no-growth boundary (ψ) were performed using Eq. 13.

|  | $\Psi= \sum_{i} \frac{{\varphi_{e}}_{i}}{2 \prod_{j\neq i} (1-{\varphi_{e}}_{j})}$ | 13 |
| --- | --- | --- |

where ψ is the growth boundary, and φ is the contributions to the interactions by each factor. The temperature term φ(T) was described by Eq. 14.

|  | $\varphi\left( T \right)=\left( \frac{T_{mes}-T}{T_{mes}-MINt} \right)^{\eta1}\left( T<T_{mes} \right)+\left( \frac{{T-T}_{mes}}{{T_{max}-T}_{mes}} \right)^{\eta2}\left( T\geq T_{mes} \right)$ | 14 |
| --- | --- | --- |

where *MINt* is the experimental minimum temperature for growth estimated from *T_min_* using this linear relationship: MINt = 5.58 + 0.71*T_min_*. The maximum enzyme stability is indicated by *T_mes_* (Table 5). η1 and η2 are shape parameters set to 3 and 2 respectively.

The φ-terms for pH and a_w_ were included in the models using Eq. 15 and 16, respectively.

|  | $\varphi\left( pH \right)={10}^{({pH}_{min}-pH)}$ | 15 |
| --- | --- | --- |

|  | $\varphi\left( a_{w} \right)={(\frac{a_{w,opt}-a_{w}}{a_{w,opt}-a_{w,min}})}^{\kappa1}$ | 16 |
| --- | --- | --- |

where *a_w,opt_* was set to 0.997, and Κ1 which is a shape parameter was set to 2.

The φ-terms for lactic and acetic acid were included using Eq. 17.

|  | $\varphi(acid)= \left( \frac{\left[ HA \right]}{{MIC}_{u}} \right)^{2}$ | 17 |
| --- | --- | --- |

The model parameter values for individual strains are listed in Table 5.

## S.7. The models by Maktabdar et al. (2025; Part 1):

This study developed two cardinal parameter models for mesophilic and psychrotolerant *B. cereus* using two strain cocktails. Each model included the effect of 11 environmental factors and the interaction between these factors. The model equations are described in Maktabdar et al. (2024). The cardinal parameters for the mesophilic and psychrotolerant models are reported in Table 6.

Table 5. Model parameters for psychrotolerant *B. cereus* strains (Le Marc et al., 2024)

| Strain | *PanC* group | *T_min_* (^o^C) | *T_opt_* (^o^C) | *T_max_* (^o^C) | *T_mes_* (^o^C) | *pH_min_* | *a_w,min_* | Acetic acid | |  | Lactic acid | |
| --- | --- | --- | --- | --- | --- | --- | --- | --- | --- | --- | --- | --- |
|  |  |  |  |  |  |  |  | *MICu* (mM) | α |  | *MICu* (mM) | α |
| MJG03 | II | 2.99 | 36.98 | 40.89 | 23.74 | 4.73 | 0.948 | 7.66 | 0.39 |  | 3.20 | 3.60 |
| ADQP403 | VI | 0.97 | 32.26 | 37.63 | 20.62 | 4.88 | 0.962 | 7.12 | 0.42 |  | 3.20 | 3.60 |
| KBAB4 | VI | 0.97 | 32.26 | 37.63 | 20.62 | 4.88 | 0.962 | 7.77 | 0.39 |  | 3.20 | 3.60 |

Table 6. Cardinal parameters for mesophilic and psychrotolerant *B. cereus* (Maktabdar et al., 2025; Part 1)

| Model parameters |  | Mesophilic model | |  | Psychrotolerant model | |
| --- | --- | --- | --- | --- | --- | --- |
|  |  | Value | Optimum |  | Values | Optimum |
| *µ_opt_* (h^-1^) |  | 2.99 | - |  | 2.12 | - |
| *µ_opt-cal_* (h^-1^) |  | - | - |  | 2.67 |  |
| *T_min_* (^o^C) |  | 7.06 | - |  | 3.80 | - |
| *T_opt_* (^o^C) |  | 39.8 | - |  | 35.1 | - |
| *T_max_* (^o^C) |  | 45.4 | - |  | 40.9 | - |
| *a_w, min_* |  | 0.955 | 0.997 (fixed) |  | 0.963 | 0.997 (fixed) |
| *pH_min_* |  | 4.75 | 6.32 |  | 4.59 | 7.53 |
| *pH_max_* |  | 9.5 (fixed) | - |  | 9.5 (fixed) | - |
| Minimum inhibitory concentrations (*MIC*s) of undissociated acids (mM) | | | | | | |
| Acetic acid (mM) |  | 15.6 | - |  | 5.66 | - |
| Benzoic acid (mM) |  | 0.36 | - |  | 0.13 | - |
| Lactic acid (mM) |  | 2.98 | ≤0.62 |  | 2.98 | ≤0.66 |
| Sorbic acid (mM) |  | 1.49 | - |  | 0.73 | - |
| Minimum inhibitory concentration (*MIC*) of total citric acid (mM) | | | | | | |
| Citric acid (mM) |  | 169 | 0 (fixed) |  | 190 | 0 (fixed) |
| Minimum inhibitory concentrations (*MIC*s) of phosphate ions (%) | | | | | | |
| Orthophosphate |  | 4.96 | - |  | 4.88 | - |
| Diphosphate |  | 2.36 | - |  | 2.21 | - |
| Triphosphate |  | 2.32 | - |  | 2.12 | - |

## References:

Carlin, F., Albagnac, C., Rida, A., Guinebretière, M.-H., Couvert, O., and Nguyen-the, C. (2013). Variation of cardinal growth parameters and growth limits according to phylogenetic affiliation in the *Bacillus cereus* group. Consequences for risk assessment. *Food Microbiol.* 33, 69–76. doi: 10.1016/j.fm.2012.08.014

Ellouze, M., Buss Da Silva, N., Rouzeau-Szynalski, K., Coisne, L., Cantergiani, F., and Baranyi, J. (2021). Modeling *Bacillus cereus* growth and cereulide formation in cereal-, dairy-, meat-, vegetable-based food and culture medium. *Front. Microbiol.* 12, 155. doi: 10.3389/fmicb.2021.639546

Le Marc, Y., Baert, L., Buss da Silva, N., Postollec, F., Huchet, V., Baranyi, J., et al. (2021). The effect of pH on the growth rate of *Bacillus cereus* sensu lato: Quantifying strain variability and modelling the combined effects of temperature and pH. *Int. J. Food Microbiol.*, 109420. doi: 10.1016/j.ijfoodmicro.2021.109420

Le Marc, Y., Petton, E., Lochardet, A., Postollec, F., and Huchet, V. (2024). Growth limits of psychrotrophic *Bacillus cereus* as a function of temperature, pH, water activity, and lactic or acetic acid. *Microb. Risk Anal.* 27–28, 100310. doi: 10.1016/j.mran.2024.100310

Maktabdar, M., Wemmenhove, E., Gkogka, E., and Dalgaard, P. (2025). Development of extensive growth and growth boundary models for mesophilic and psychrotolerant *Bacillus cereus* in dairy products (Part 1). Front. Microbiol. 16:1553885. doi: 10.3389/fmicb.2025.1553885.

Ölmez, H. K., and Aran, N. (2005). Modeling the growth kinetics of *Bacillus cereus* as a function of temperature, pH, sodium lactate and sodium chloride concentrations. *Int. J. Food Microbiol.* 98, 135–143. doi: 10.1016/j.ijfoodmicro.2004.05.018

Zwietering, M. H., de Wit, J. C., and Notermans, S. (1996). Application of predictive microbiology to estimate the number of *Bacillus cereus* in pasteurised milk at the point of consumption. *Int. J. Food Microbiol.* 30, 55–70. doi: 10.1016/0168-1605(96)00991-9
